# Surgically treated purulent pericarditis induced by ingested fish bone: a case report

**DOI:** 10.1186/s44215-023-00113-7

**Published:** 2023-11-16

**Authors:** Yoshifumi Itoda, Toshiya Fukushima, Shuhei Kawamoto, Motoharu Shimozawa, Retsu Tateishi, Fumiya Haba, Shunya Ono, Yoshinori Nakahara, Takeyuki Kanemura

**Affiliations:** Department of Cardiovascular Surgery, IMS Katsushika Heart Center, 3-30-1, Katsushika, Tokyo, 124-0006 Japan

**Keywords:** Ingested foreign body, Esophageal perforation, Purulent pericarditis

## Abstract

**Background:**

Aspiration of fish bones is common, but perforation of the gastrointestinal tract is very rare. Once perforation occurs, fatal complications such as mediastinitis and cardiac tamponade can occur. Here, we present a case of acute pericarditis due to perforation of a fish bone.

**Case presentation:**

A 66-year-old woman was referred to our hospital with shortness of breath. Blood tests showed high C-reactive protein, and contrast-enhanced computed tomography showed a large amount of pericardial fluid as well as a foreign body with high bone density in the pericardial sac. Upper gastrointestinal endoscopy showed no evidence of penetration of the esophageal or gastric mucosa. Emergency open chest surgery was performed, and the pericardial sac was filled with copious amounts of pus. The fish bone-like foreign body was found to penetrate the pericardial membrane from the diaphragmatic side. The foreign body was removed, a drainage tube was placed, and the chest was closed. After 2 weeks of postoperative antibiotics, the patient was discharged from the hospital in stable general condition. Three months after the surgery, the patient had no recurrence of pericarditis.

**Conclusions:**

We reported a rare case of gastrointestinal perforation by a fish bone, resulting in pericardium, which was treated by surgical drainage.

## Background

Perforation of the gastrointestinal tract due to aspiration of a foreign body can present with a variety of clinical manifestations, depending on the site. Pericarditis caused by aspiration of fish or chicken bones is very rare, but can be a serious condition, so prompt diagnosis and appropriate treatment are important.

## Case presentation

**Fig. 1:**
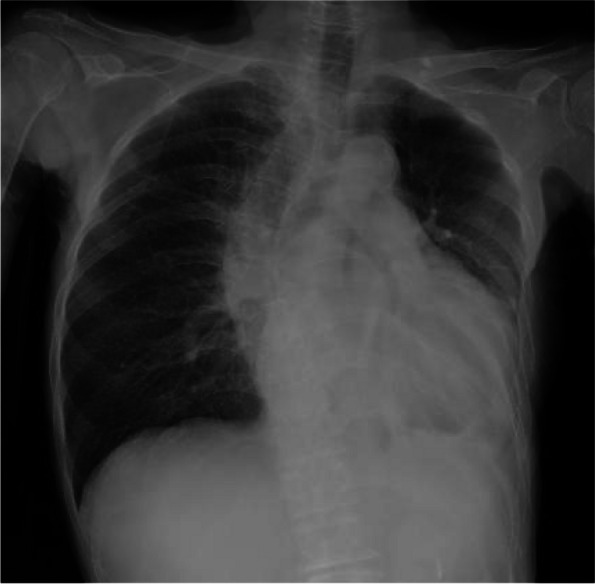
Chest X-ray on admission. Chest X-ray showed scoliosis and cardiac enlargement

**Fig. 2:**
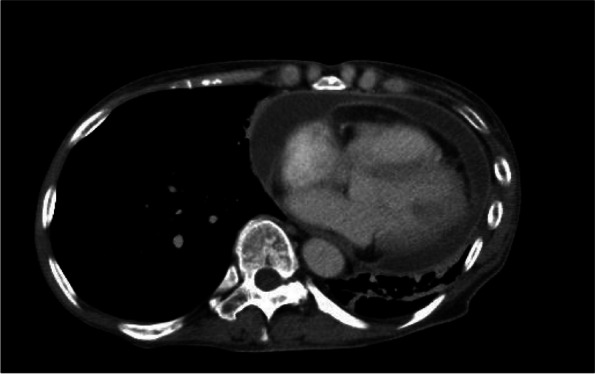
Chest CT on admission. Chest CT showed massive pericardial effusion with thickening of the pericardium

**Fig. 3:**
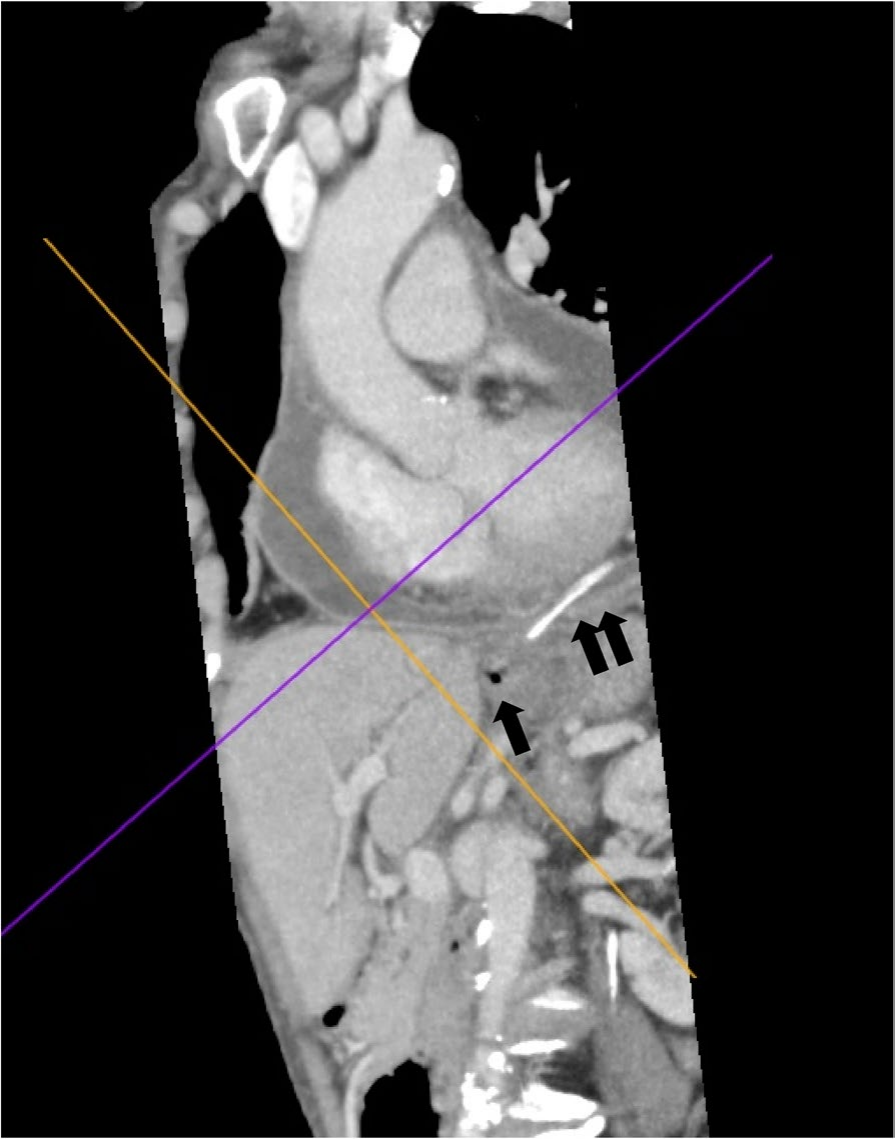
Foreign body found in pericardiac sac. A 4-cm-long needle-shaped foreign body was seen in the pericardial sac (double arrow). The tip did not reach the gastrointestinal tract (single arrow)

**Fig. 4:**
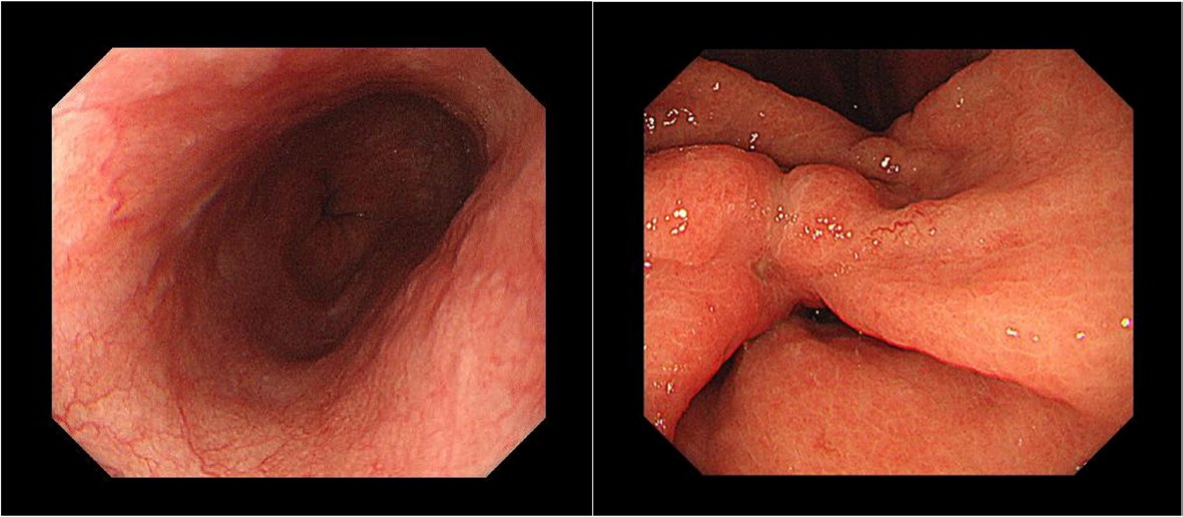
Preoperative gastrointestinal endoscopy. The esophageal mucosa showed an ulcer scar on the small arm side of the gastric body without findings of perforation

A 66-year-old woman presented to a local clinic complaining of shortness of breath on exertion that lasted approximately 1 week. Suspecting pneumonia, she was treated with oral antibiotics, but her condition did not improve, and she was referred to our hospital because a computed tomography (CT) revealed a large amount of pericardial effusion. She presented with fever and hypotension, and blood tests showed a C-reactive protein of 17.8 mg/dl and white blood cell elevated to 23500 μl. Chest X-ray showed scoliosis and cardiac enlargement (Fig. 1). Electrocardiogram showed a sinus rhythm of 88 bpm and no specific S-T changes. Echocardiography showed normal left ventricular contractility and no regional wall motion abnormalities. A circumferential pericardial effusion was observed, some of which appeared to be fibrinous tissue. Ventricular inflow suggested the presence of diastolic dysfunction. There was no evidence of valvular disease. Contrast-enhanced CT showed thickened pericardial membranes and a large amount of pericardial effusion, suggesting inflammation (Fig. 2). A needle-shaped foreign body showing high bone density was observed on the diaphragmatic side of the pericardiac sac. It appeared to penetrate the diaphragm from the esophagogastric junction and reach the pericardial sac, but the lower tip did not reach the digestive tract (Fig. 3). An upper gastrointestinal endoscopy (GIE) was performed to determine the origin of the foreign body. The results showed only an ulcer scar on the small arm of the gastric body and no abnormal findings on the esophageal mucosa (Fig. 4), which did not prove that the foreign body originated from the digestive tract. The patient was asked about her feeding history, but she did not recall ingestion of any foreign objects, including fish or chicken bones.

**Fig. 5:**
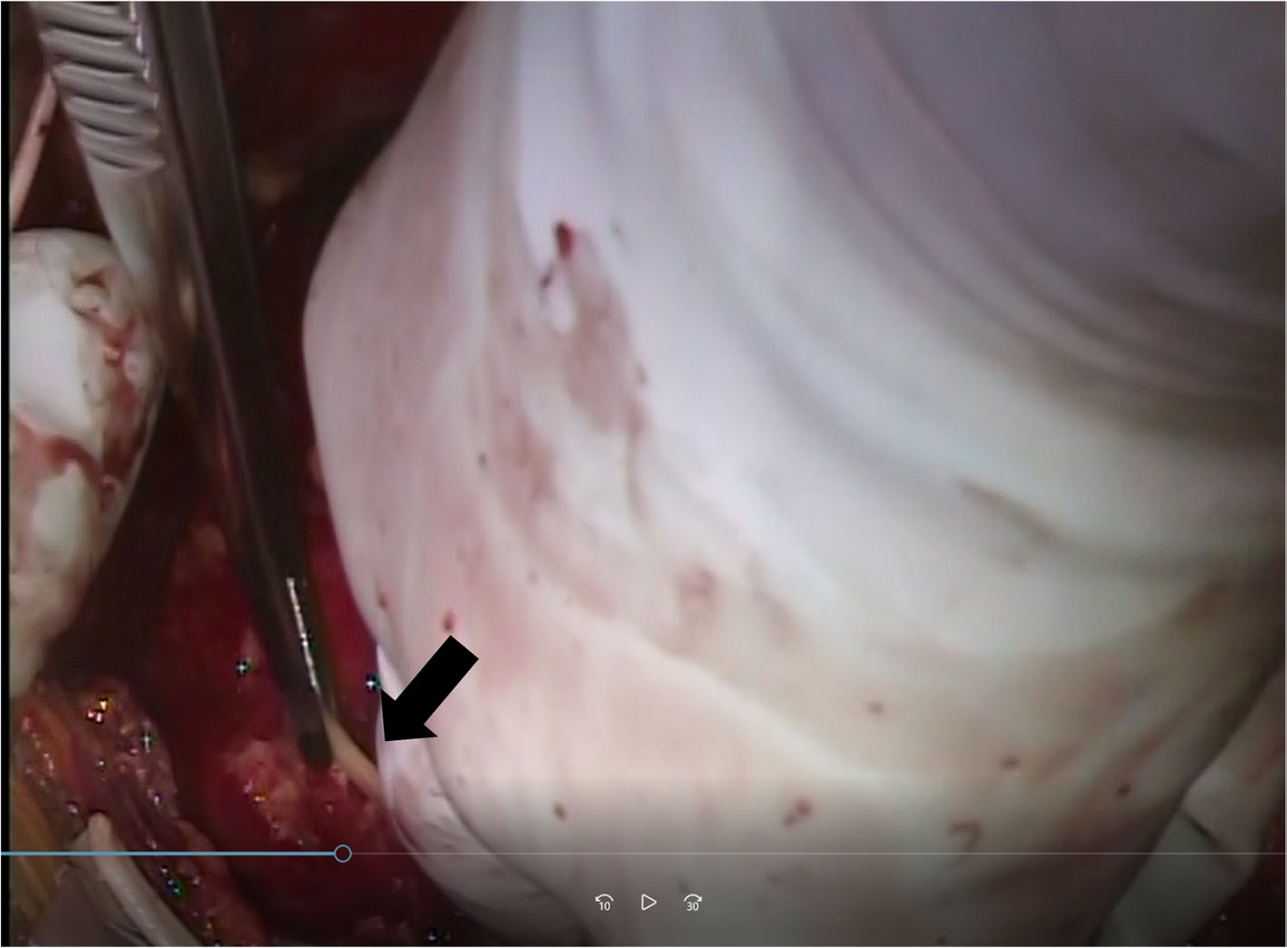
Operative findings. The fish bone-like foreign body (arrow) was found on the diaphragmatic side of the pericardial sac, and the caudal side penetrated the diaphragm. The pericardial sac was filled with infectious pus

**Fig. 6:**
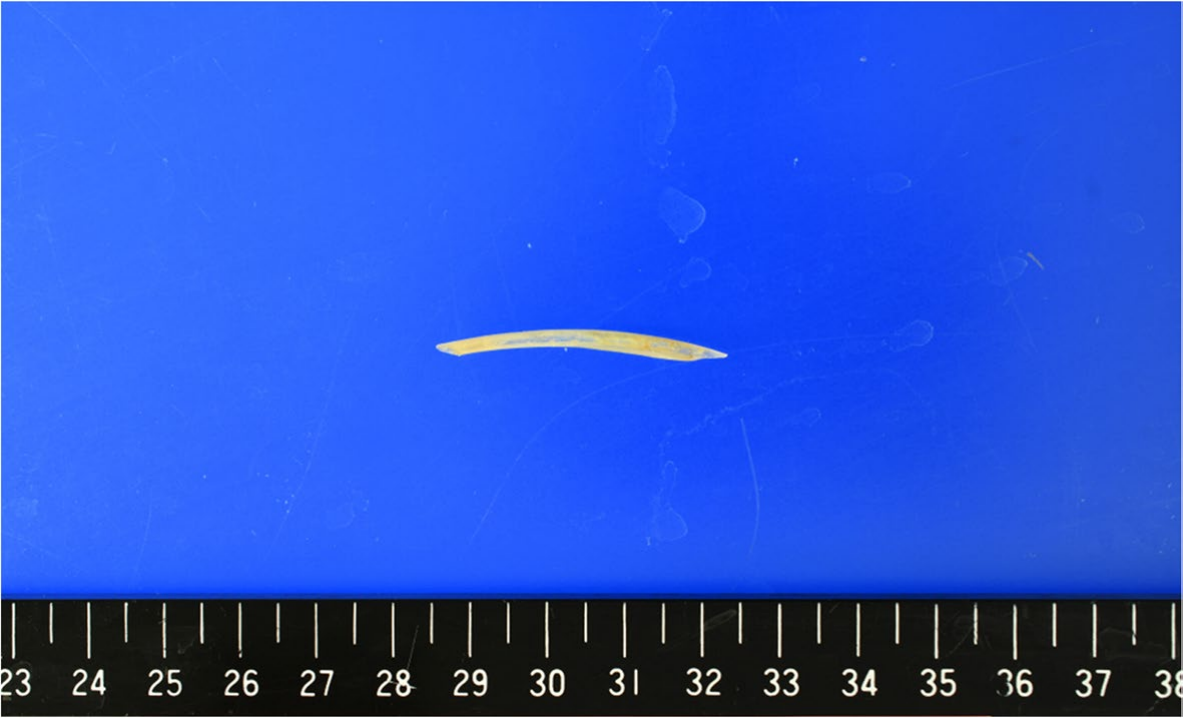
The removed fish bone. Approximately 40 mm of removed fish bone

We diagnosed the patient with pericarditis caused by a foreign body in the pericardial sac and performed emergency surgery. After a median sternotomy, the pericardial sac was opened, and a large amount of purulent pericardial effusion was observed. A culture test of the pericardial fluid submitted at that time later detected *Enterobacter aerogenes*. When the heart was lifted, a needle-shaped foreign body that appeared to be a fish bone was observed on the diaphragmatic side of the pericardial sac (Fig. 5). Removal of the foreign body revealed a small defect in the pericardial sac membrane, which was sutured closed. The pericardial sac was flushed with a sufficient volume of saline solution, a drain was placed, and the chest was closed. During surgery, the sternal cut edge was covered with a sheet of cut-open rubber gloves to isolate it from contaminated pericardial fluid and prevent sternal osteomyelitis. The foreign body removed was later identified as fish bones by pathological examination (Fig. 6).

**Fig. 7:**
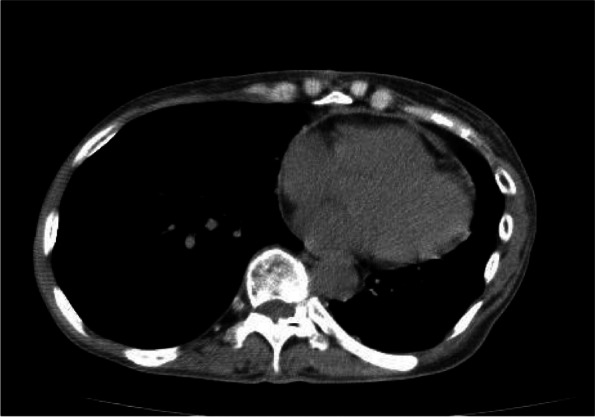
Chest CT 3 months after surgery. The patient had no recurrence of pericarditis at 3 months postoperatively

Postoperatively, based on the culture results, antibiotic therapy with ceftriaxone was continued for 2 weeks, and the patient was discharged with a stable general condition. In the outpatient setting, the patient underwent blood draws and CT over time. Three months postoperatively, there was no evidence of an elevated inflammatory response or pericardial effusion (Fig. 7).

## Discussion and conclusions

Although ingestion of foreign bodies such as fish bones or meat bones is a very common event, gastrointestinal perforation is very rare and is known to cause serious complications [1–4]. Because the gastrointestinal tract is in contact with various organs, various complications can occur depending on where the bone is trapped, including adenitis, cardiac tamponade [1], pericarditis [2–4], pyothorax, liver abscess [5], and pancreatic abscess [6]. Often, the patient’s dietary history is often ambiguous, and often misdiagnosed as acute myocardial infarction, pericarditis, pneumonitis pleurisy, or other abdominal organ diseases. Therefore, suspicion and appropriate testing, including CT and GIE, are important for correct diagnosis. In the current case, GIE revealed no evidence of gastrointestinal perforation scarring, but the shape and location of the foreign body made esophageal perforation by a fish bone the most suspicious. In a study by Yang et al. summarizing esophageal perforation from benign disease, they reported that 118 of 135 cases were due to ingestion of a foreign body, 78 of which were fish bones [7].

Mediastinitis, including pericarditis, is one of the most serious complications of esophageal perforation by a foreign body, and the basis of treatment is the removal of the foreign body and drainage of the contaminated pericardial fluid and administration of antibiotics. Kim et al. reported the death of 3 of 39 surgically treated cases due to exacerbation of mediastinitis [8]. Chikuie et al. reported a case of surgical drainage for pericarditis that recurred and required additional puncture drainage. In that case, the esophageal perforation was small but open [1]. In our case, the perforation on the esophageal side had already healed and closed, there were no abscesses formed in the mediastinum other than in the pericardial sac. Omentopexy was considered according to the treatment of mediastinitis, but based on the above findings, the possibility of exacerbation of infection was judged to be low, so only removal of the foreign body, lavage of the pericardial sac, and placement of a drain tube were performed. We prevented contamination by covering the sternal fragment with rubber gloves during surgery, resulting in an uncomplicated recovery. Although we have not found any papers that mention the duration of postoperative antibiotic administration, in our case, effective antibiotics based on the culture results were administered for a sufficiently long period of time.

In conclusion, we successfully treated a case of purulent pericarditis caused by the ingestion of a fish bone by surgical treatment. Prompt diagnosis through appropriate testing and subsequent treatment was considered important.

## Data Availability

Not applicable.

## Abbreviations

CT: Computed tomography
GIE: Gastrointestinal endoscopy
